# Resveratrol Ameliorates Cardiac Remodeling in a Murine Model of Heart Failure With Preserved Ejection Fraction

**DOI:** 10.3389/fphar.2021.646240

**Published:** 2021-06-10

**Authors:** Liyun Zhang, Juan Chen, Lianhua Yan, Qin He, Han Xie, Manhua Chen

**Affiliations:** Department of Cardiology, The Central Hospital of Wuhan, Tongji Medical College, Huazhong University of Science and Technology, Wuhan, China

**Keywords:** resveratrol, heart failure with preserved ejection fraction, inflammation, macrophage polarization, oxidative stress

## Abstract

**Objective:** Accumulating evidence suggested that resveratrol (RES) could protect against adverse cardiac remodeling induced by several cardiovascular diseases. However, the role of RES in the setting of heart failure with preserved ejection fraction (HFpEF) and the underlying mechanisms of its action remain understood. This study was to determine whether RES could ameliorate HFpEF-induced cardiac remodeling and its mechanisms.

**Methods:** *In vivo*, C57BL/6 mice served as either the sham or the HFpEF model. The HFpEF mice model was induced by uninephrectomy surgery and d-aldosterone infusion. RES (10 mg/kg/day, ig) or saline was administered to the mice for four weeks. *In vitro*, transforming growth factor β1 (TGF-β1) was used to stimulate neonatal rat cardiac fibroblasts (CFs) and Ex-527 was used to inhibit sirtuin 1 (Sirt1) in CFs. Echocardiography, hemodynamics, western blotting, quantitative real-time PCR, histological analysis, immunofluorescence, and ELISA kits were used to evaluate cardiac remodeling induced by HFpEF. Sirt1 and Smad3 expressions were measured to explore the underlying mechanisms of RES.

**Results:** HFpEF mice developed left ventricular hypertrophy, preserved ejection fraction, diastolic dysfunction, and pulmonary congestion. Moreover, HFpEF mice showed increased infiltration of neutrophils and macrophages into the heart, including increased interleukin (IL)-1β, IL-6, and TNF-α. We also observed elevated M1 macrophages and decreased M2 macrophages, which were exhibited by increased mRNA expression of M1 markers (iNOS, CD86, and CD80) and decreased mRNA expression of M2 markers (Arg1, CD163, and CD206) in HFpEF hearts. Moreover, HFpEF hearts showed increased levels of intracellular reactive oxygen species (ROS). Importantly, HFpEF mice depicted increased *collagen-I* and *-III* and TGF-β mRNA expressions and decreased protein expression of phosphorylated endothelial nitric-oxide synthase (p-eNOS). Results of western blot revealed that the activated TGF-β/Smad3 signaling pathway mediated HFpEF-induced cardiac remodeling. As expected, this HFpEF-induced cardiac remodeling was reversed when treated with RES. RES significantly decreased Smad3 acetylation and inhibited Smad3 transcriptional activity induced by HFpEF *via* activating Sirt1. Inhibited Sirt1 with Ex-527 increased Smad3 acetylation, enhanced Smad3 transcriptional activity, and offset the protective effect of RES on TGF-β–induced cardiac fibroblast–myofibroblast transformation in CFs.

**Conclusion:** Our results suggested that RES exerts a protective action against HFpEF-induced adverse cardiac remodeling by decreasing Smad3 acetylation and transcriptional activity *via* activating Sirt1. RES is expected to be a novel therapy option for HFpEF patients.

## Introduction

Heart failure with preserved ejection fraction (HFpEF) has been gradually increasing due to increasing aging population; it was reported that HFpEF now accounts for nearly half (39–72%) of all heart failure patients (Owan et al., 2006; Udelson, 2011). Adverse cardiac remodeling and diastolic dysfunction are hallmarks of HFpEF, which are characterized by left ventricle (LV) hypertrophy, elevated stiffness, and increased filling pressure (Schwarzl et al., 2016), but the underlying mechanisms remain unclear. Recently, HFpEF is regarded as a systemic syndrome affected by risk factors and comorbidities. The underlying mechanism is not fully elucidated and strategies to cure this global puzzle are limited. Myocardial inflammation, oxidative stress, and coronary endothelial dysfunction which lead to stiffness of cardiomyocytes, cellular hypertrophy, and enhanced myocardial fibrosis are purported to be the potential mechanisms of HFpEF (Esposito et al., 2017; van der Pol et al., 2018). Theoretically, finding molecules that suppress HFpEF-induced cardiac inflammation, oxidative stress, and myocardial fibrosis would be of great benefit with respect to the therapy of HFpEF.

**FIGURE 1:**
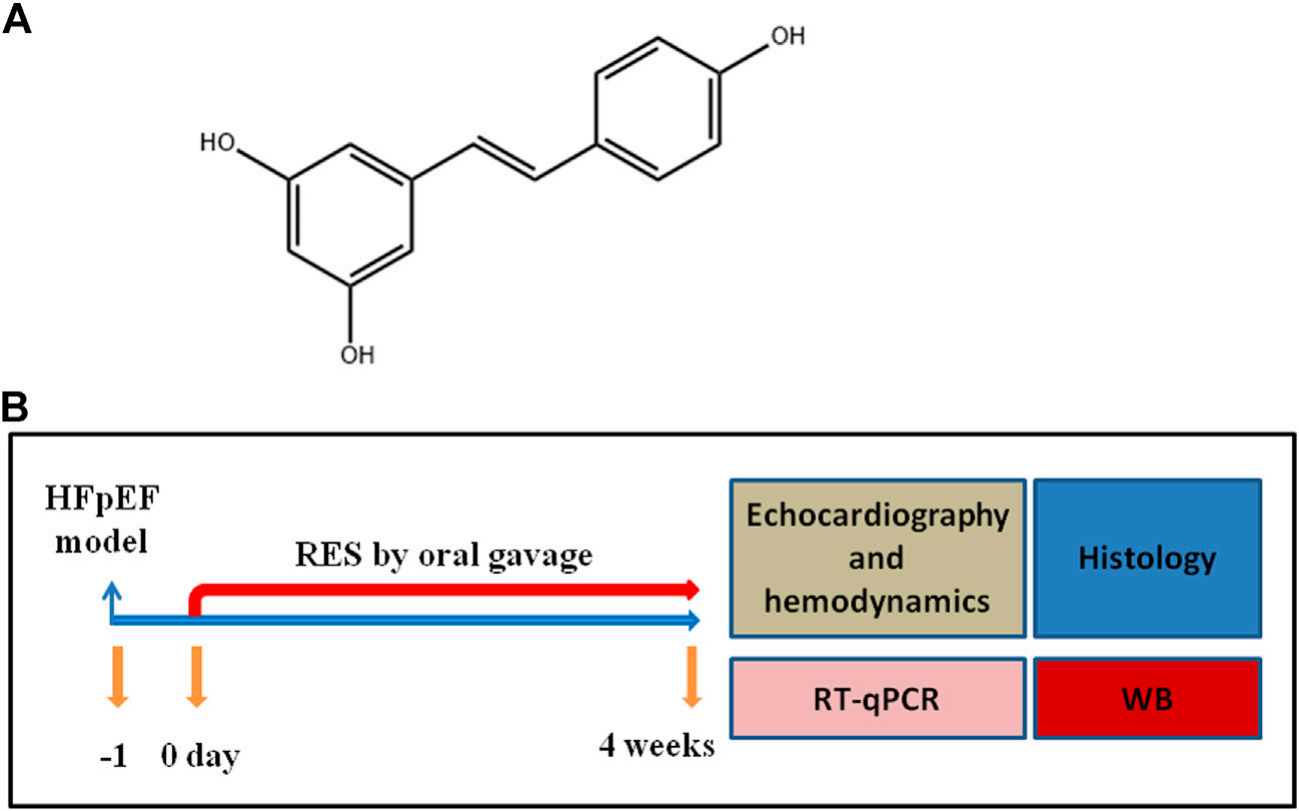
**(A)** Structure of RES. **(B)** Experimental protocol for HFpEF mice administered vehicle or resveratrol (RES).

Resveratrol (3,5,4′-trihydroxystilbene) (RES) (Figure 1A) is a natural phytoalexin found in a wide variety of plant species and present in varying concentrations in red wines (Dolinsky and Dyck, 2011). Accumulating evidence revealed that RES was involved in several pathological processes including cardiac hypertrophy (Fan et al., 2016), myocardial infarction (Penumathsa et al., 2007), and heart failure (Sung et al., 2017). RES was reported to be a good candidate agent for several cardiovascular diseases owing to its protective action against cardiac fibrosis, oxidation, inflammation, and platelet oxidation (Delmas et al., 2005). However, the effect of RES on HFpEF and the precise mechanism are poorly understood.

Sirtuin 1 (Sirt1) deacetylase, a class III histone deacetylase, could deacetylate various proteins (Kume et al., 2017). Recently, Sirt1 was reported to play an important role in isoproterenol (ISO)-induced oxidative stress, endoplasmic reticulum stress, and cardiac fibrosis (Li et al., 2018). Sirt1 was found to interfere with cardiac fibroblasts by interacting with Smad3 (Liu et al., 2019). Besides, Sirt1 was also associated with cardiac hypertrophy and inflammation (Planavila et al., 2011). Emerging studies reported that decreased deacetylase activity and protein expression of Sirt1 were found in hearts under pathological stimulation and that activated Sirt1 could protect against cardiac fibrosis by decreasing Smad3 acetylation and reducing Smad3 transcriptional activity in hearts (Cappetta et al., 2016; Ma et al., 2017; Li et al., 2018). RES, an activator of Sirt1, could prevent cardiac fibrosis and generation of reactive oxygen species (ROS) in cardiomyocytes through Sirt1 activation (Li et al., 2013).

Accordingly, we theorized that RES can effectively protect against HFpEF-induced cardiac inflammation, oxidation, and fibrosis by activating Sirt1.

## Materials and Methods

### Reagents

RES was purchased from Sigma (#R5010, St. Louis, MO, United States), and d-aldosterone was purchased from Sigma (#706035, St. Louis, MO, United States). Ex-527 was purchased from Sigma (#E7034).

### Animals

Male C57BL/6 mice (8- to 10-week-old) were supplied by the animal experiment center of Tongji Medical College, Huazhong University of Science and Technology. Mice were allowed 1 week to acclimatize to a stable environment before experiments began. Mice were individually housed in plastic cages with bedding, ad libitum food, and tap water. The cages were maintained at a temperature of 22 ± 2°C and a 12:12 h light/dark cycle. Mice were randomly assigned to four groups: sham with vehicle (sham-vehicle), sham with RES (sham-RES), HFpEF with vehicle (HFpEF-vehicle), and HFpEF with RES (HFpEFRES). All mice underwent uninephrectomy and received a continuous infusion of either saline (sham) or d-aldosterone (0.15mg/h) (HFpEF) for 4 weeks *via* osmotic mini-pumps (Alzet, Durect Corp., Cupertino, CA, United States) (Tanaka et al., 2014). Twenty-4 hours after the surgery, mice were administered RES (10 mg/kg/day) by oral gavage for the duration of 4 weeks (Gupta et al., 2014) (Figure 1B). All experimental procedures were according to the Guidelines for the Care and Use of Laboratory Animals published by the United States National Institutes of Health and were approved by the Animal Care and Use Committee of Tongji Medical College, Huazhong University of Science and Technology (Approval Number: 20191808).

### Echocardiography and Hemodynamics Analysis

Isoflurane (1.5%) was used to anesthetize; then, echocardiography was conducted using a MyLab 30CV ultrasound machine with a 10 MHz linear array ultrasound transducer. M-mode images of the left ventricle at the papillary muscle level were recorded, and then left ventricular ejection fraction (EF) and fractional shortening (FS) were measured (Yang et al., 2020). Hemodynamic parameters were analyzed with a Millar catheter transducer (SPR-839; Millar Instruments, Houston, TX, United States). After stabilization for 30 min, the left ventricular systolic function and diastolic function indicators were recorded continuously with an ARIA pressure–volume conductance system coupled with a PowerLab/4SP A/D converter. The data were analyzed using LabChart 7 software, according to the 2017 ACC/AHA/HFSA Guideline for the Management of Heart Failure; those mice with EF lower than 45% were excluded from the experiment (Yancy et al., 2017), resulting in the exclusion of three mice.

### Histological Analysis

Hearts were isolated and arrested in 10% KCl solution. Then, after fixation with 4% paraformaldehyde for 5 days, the hearts were embedded in paraffin and sliced into approximately 5 µm sections. Subsequently, the sections were stained with wheat germ agglutinin (WGA) for the determination of the cardiomyocyte size; 10 different areas per heart were evaluated.

### Immunofluorescence

For immunofluorescence detection, the sections with a thickness of 10 μm were incubated with the primary antibody against Ly6G (#MAB1037, R&D Systems), F4/80 (#MAB5580, R&D Systems), CD80 (#AF740, R&D Systems), and CD206 (#AF2535, R&D Systems); afterward, sections were incubated with FITC-conjugated anti-rabbit whole IgG and Texas Red–conjugated anti-mouse whole IgG. The stained sections were observed and photographed under a microscope (×200) of 20 random fields of view of each heart sample.

### Inflammatory Cytokine Measurement

The prepared serum was assayed for IL-1β (#EMC001b.96), IL-6 (#YIF-LF-EK0270), and TNF-α (#EMC102a.96) using the Quantikine Sandwich ELISA Kit following the manufacturer’s instructions (NeoBioscience Technology Co., China).

### Fibrosis Quantification

Briefly, hearts were transversely cut close to the papillary muscles, fixed in 10% phosphate-buffered formalin, and embedded in paraffin. The paraffin-embedded hearts were sliced into 5 μm sections that were stained with picrosirius red (PSR) to quantify cardiac fibrosis. The stained sections were observed and photographed under a microscope (×200) of 15 random fields of view of each heart sample. The left anterior free-wall fields were analyzed and averaged to calculate the collagen fibrosis in each heart.

### Reactive Oxygen Species Detection

To detect ROS, DHE staining was conducted. Briefly, cryosections of fresh heart samples were stained with DHE (10 μM, #S0063, Beyotime, China) for 30 min at 37°C to detect ROS production. Pictures were taken with an OLYMPUS DX51 fluorescence microscope (Tokyo, Japan). Furthermore, fresh heart samples were homogenized on ice and centrifuged at 3,000 rpm for 15 min. After that, the supernatants were collected to measure the activities of superoxide dismutase (SOD, #S0109, Beyotime, China) and catalase (CAT, #S0082, Beyotime, China) and the content of glutathione (GSH, #S0053, Beyotime, China) by commercial kits according to the manufacturer’s instructions. The stained sections were observed and photographed under a microscope (×200) of 20 random fields of view of each heart sample.

### Neonatal Rat Cardiac Fibroblasts’ Culture and Treatment

Neonatal rat cardiac fibroblasts (CFs) were isolated from the newborn Sprague Dawley rats (1–3 days) as previously reported (Li et al., 2018). Briefly, the left ventricles of neonatal rats were minced and digested with collagenase II (50 U ml^−1^) and trypsin (0.1%). Then, cells were obtained and plated for 1.5 h at 37°C until the CFs adhered to the wall of the plate. After discarding the pre-seeding medium containing cardiomyocytes and unattached cells, the relatively pure CFs were collected. CFs were then cultured in DMEM/F12 (Gibco, Carlsbad, CA, United States) with 10% fetal bovine serum (FBS, Gibco, Carlsbad, CA, United States) at 37°C in a humidified incubator with 5% CO_2_. All CFs were treated within three passage cultures, but only CFs prior to the third passage could be used in our study. After serum starvation for 24 h and synchronization, CFs were randomly treated with TGF-β (10 ng/ml, #240-B-010, R&D, MN, United States) and RES (25 μm) for 24 h. The Sirt1 expression in CFs was blocked by Ex-527 (10 µM).

### Quantitative Real-Time PCR

Total RNA was prepared using TRIzol reagent (Invitrogen) from heart samples or cell lysates. Two micrograms of RNA were reverse transcribed into cDNA with random primers using a Transcriptor First Strand cDNA Synthesis Kit. Real-time PCR was performed using LightCycler 480 SYBR Green 1 Master Mix. The PCR conditions were as follows: 95°C for 10 min, followed by 40 cycles of 95°C for 5 s, 60°C for 10 s, and 72°C for 15 s and a final step at 72°C for 15 s. The level of each gene was measured in triplicate in at least three independent experiments. Calculations of relative mRNA expression levels were conducted using the ΔΔCT method. GAPDH was used as an internal reference. The mRNA data were normalized to GAPDH and the sham-vehicle and PBS-treated groups. All primer details are listed in Table 1.

**TABLE 1:** Mouse primers for RT-PCR.

Gene	Forward primers	Reverse primers
ANP	GGA​GCA​AAT​CCC​GTA​TAC​AGT​G	CTC​TGA​GAC​GGG​TTG​ACT​TCC
BNP	TCA​AAG​GAC​CAA​GGC​CCT​AC	CTA​AAA​CAA​CCT​CAG​CCC​GTC
β-MHC	GAT​GGT​GAC​ACG​CAT​CAA​CG	CCA​TGC​CGA​AGT​CAA​TAA​ACG
Collagen-I	CCG​TGA​CCT​CAA​GAT​GTG​CC	GAA​CCT​TCG​CTT​CCA​TAC​TCG
Collagen-III	GAC​CTC​CTG​GAA​AAG​ATG​GAT​C	AAA​TCC​ATT​GGA​TCA​TCC​CC
TGF-β	GTG​GCT​GAA​CCA​AGG​AGA​CG	AGG​TGT​TGA​GCC​CTT​TCC​AG
iNOS	CGA​AAC​GCT​TCA​CTT​CCA​A	TGA​GCC​TAT​ATT​GCT​GTG​GCT
CD86	GCT​TCA​GTT​ACT​GTG​GCC​CT	TGT​CAG​CGT​TAC​TAT​CCC​GC
CD80	GGC​CTG​AAG​AAG​CAT​TAG​CTG	GAG​GCT​TCA​CCT​AGA​GAA​CCG
Arg1	AAC​ACG​GCA​GTG​GCT​TTA​ACC	GGT​TTT​CAT​GTG​GCG​CAT​TC
CD163	TCC​ACA​CGT​CCA​GAA​CAG​TC	CCT​TGG​AAA​CAG​AGA​CAG​GC
CD206	CAG​GTG​TGG​GCT​CAG​GTA​GT	TGT​GGT​GAG​CTG​AAA​GGT​GA
Sirt1	TGT​GTC​ATA​GGT​TAG​GTG​GTG​A	AGC​CAA​TTC​TTT​TTG​TGT​TCG​TG
GAPDH	CGC​TAA​CAT​CAA​ATG​GGG​TG	TTG​CTG​ACA​ATC​TTG​AGG​GAG

### Western Blotting Analysis

Total protein was extracted and separated on SDS-PAGE (AS1086, ASPEN). Then, proteins were transferred onto PVDF membranes (EMD Millipore, Billerica, MA). The membranes were incubated overnight at 4°C with the primary antibody of IL-1β (#ab234437, Abcam), IL-6 (#ab229381, Abcam), TNF-α (#ab205587, Abcam), TGF-β1 (#ab215715, Abcam), p-eNOS (#ab230158, Abcam), eNOS (#ab76198, Abcam), Sirt1 (#ab110304, Abcam), and GAPDH (#ab37168, Abcam). The membranes were then incubated for 2 h at room temperature with HRP-conjugated secondary antibodies (#AS1107, ASPEN). The western blot film images were scanned and analyzed using ImageJ (NIH, Bethesda, MD). The total protein levels were normalized to GAPDH and the sham-vehicle group.

### Immunoprecipitation and Assessment of Smad3 Acetylation

Immunoprecipitation analysis was performed as described previously (Huang et al., 2014). Heart tissues and CFs were homogenized and lysed on ice for 15 min in hypotonic lysis buffer containing protease inhibitor mixture. The lysates were clarified under centrifugation at 12,000 g at 4°C for 15 min. A 500 μg sample of the protein extract was incubated with mouse anti-Smad3 antibodies (#ab227223, Abcam) for 4 h at 4 °C and then with an appropriate protein A agarose or protein G agarose (Beyotime Company, Shanghai, China) overnight at 4°C while shaking. The beads were washed three times, solubilized in SDS sample buffer, and then submitted to western blot analysis with rabbit anti-acetylated lysine (#AcK-103, Cell Signaling Technology) or anti-Smad3 (#ab227223, Abcam) antibodies.

### Sirtuin 1 Deacetylase Activity Assay

Equal amounts of total protein were used for each experimental condition. The reagents from a Sirt1 Assay Kit (#ab156065, Abcam) were used according to the manufacturer’s instructions. Sirt1 deacetylase activity was measured using fluorescence intensity signals at 450 nm (excitation, 360 nm) using a microplate fluorimeter. Experimental values are represented as activity relative to that observed in the sham-vehicle group or PBS group.

### Transcriptional Activity of Smad3

The transcriptional activity of Smad3 was measured using a luciferase reporter gene assay. Briefly, the CFs were transfected with a firefly luciferase reporter plasmid Smad3-CAGA-Luc mixed with an internal control pRL-TK vector (Promega) as described previously (Huang et al., 2014). The transfection procedure was performed for 6 h using Lipofectamine 2000 (Invitrogen) according to the manufacturer’s instructions. After transfection and serum starving for 24 h, TGF-β (10 ng/ml) and RES (25 μM) were then added to the cells for 24 h, and the cells were then treated with Ex-527 (10 μM) for 24 h. A dual luciferase assay kit (#RG089S, Beyotime, China) was used according to the manufacturer’s protocol to quantify luciferase activity. The relative luciferase activity was defined as Smad3 reporter firefly luciferase activity adjusted by Renilla luciferase activity expressed from pRL-TK. The data are presented as fold induction compared with those in the control group shown as the mean ± SD of three assays that were performed in triplicate.

### Statistical Analysis

All data in the tables and figures are expressed as mean ± SD and were analyzed using GraphPad Prism 8.0 software. Differences between two groups were assessed using Student’s *t*-test or chi-square test. The differences between multiple groups were analyzed with two-way analysis of variance (ANOVA) with Tukey’s test. *p* < 0.05 was considered statistically significant.

## Results

### Resveratrol Ameliorated Heart Failure With Preserved Ejection Fraction Phenotype

**FIGURE 2:**
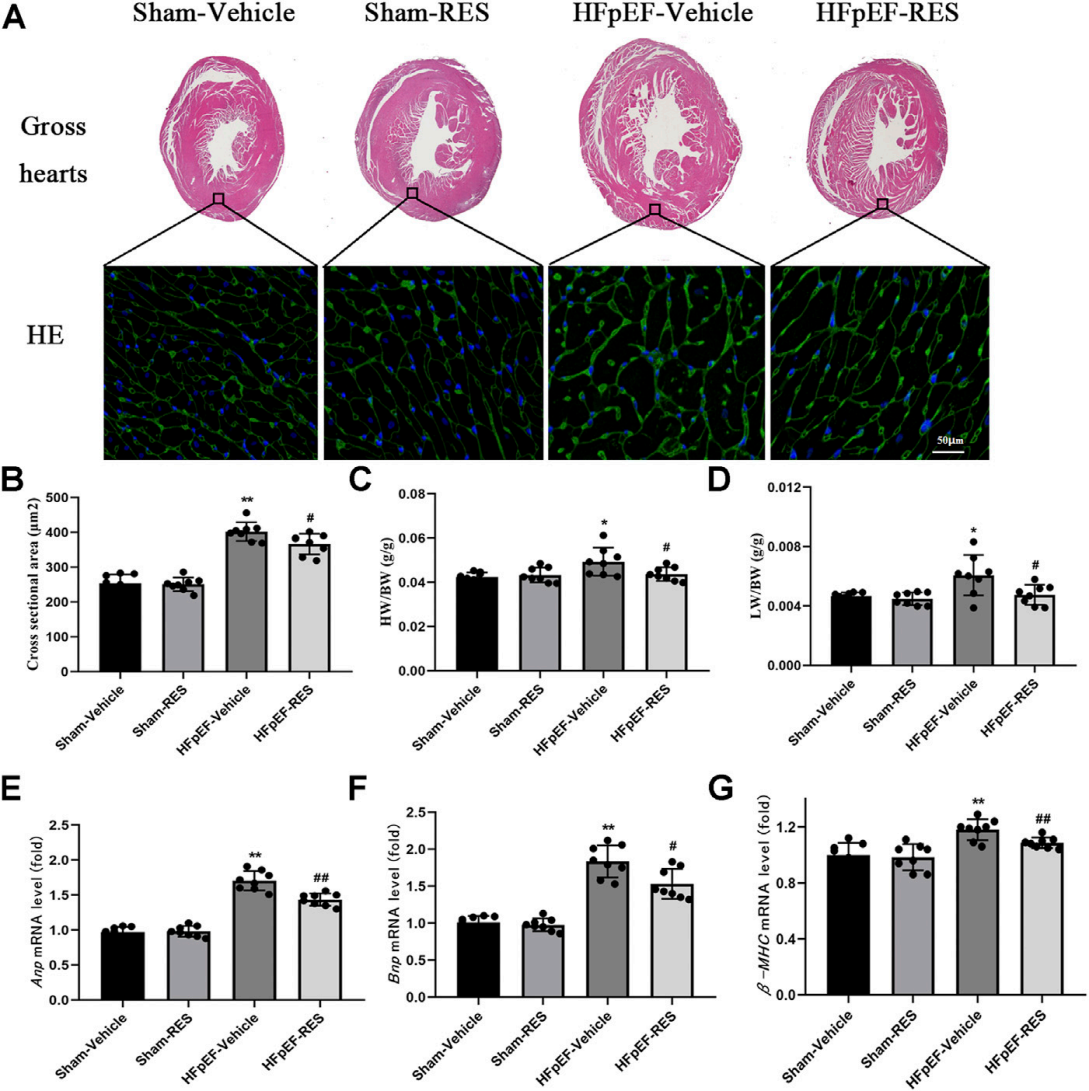
Effect of RES on the HFpEF phenotype. **(A)** Representative images of gross hearts and WGA-stained heart sections. **(B)** Quantitative results of the left ventricular cross-sectional area (*n* = 8 per group). **(C)** HW/BW ratio (*n* = 8 per group). **(D)** LW/BW ratio between the four groups (*n* = 8 per group). **(E–G)** Results of *ANP*, *BNP*, and *β-MHC* mRNA levels (*n* = 8 per group). *p < 0.05 vs. sham-vehicle group. **p < 0.01 vs. sham-vehicle group. #*p* < 0.05 vs. HFpEF-vehicle group. ##*p* < 0.01 vs. HFpEF-vehicle group.

Firstly, we examined if the RES administration caused a change in HFpEF phenotype. Left ventricular hypertrophy (LVH) is an important characteristic of HFpEF. We initially checked the LVH in HFpEF mice which was measured by cardiomyocytes’ size; WGA staining showed that the cross-sectional area of LV cardiomyocytes in the HFpEF-vehicle group was significantly larger than that in the sham-vehicle group (*p* < 0.05, Figure 2A), and RES administered significantly decreased the cross-sectional area of LV cardiomyocytes in the HFpEF-RES group when compared with the HFpEF-vehicle group (*p* < 0.05, Figures 2A,B). As shown in Figure 2C, the heart weight-to-body weight (HW/BW) ratio was significantly greater in the HFpEF-vehicle group than the sham-vehicle group (*p* < 0.05). RES treatment significantly decreased the HW/BW ratio in the HFpEF-RES group than the HFpEF-vehicle group (*p* < 0.05) and obviously reversed HFpEF-induced LVH, which was further verified by the mRNA levels of LVH markers (ANP, BNP, and β-MHC, Figures 2E–G). Altogether, these clues suggested that RES can offset HFpEF-induced LVH. Preserved ejection fraction and diastolic dysfunction are another two vital features of HFpEF (Pfeffer et al., 2019). We then investigated the role of RES in HFpEF-induced preserved ejection fraction and diastolic dysfunction by conducting echocardiography and pressure–volume analysis. As expected, HFpEF mice showed a normal LVEF and LVFS (Table 2). Furthermore, HFpEF mice also depicted diastolic dysfunction, which was shown by increased dp/dtmin, end-diastolic pressure–volume relationship (EDPVR)-k (constant of an exponential curve fit of the EDPVR), and tau index when compared with those in sham-vehicle mice (*p* < 0.05, Table 2); RES treatment significantly reversed HFpEF-induced diastolic dysfunction, which was shown by decreasing the increase in dp/dtmin, EDPVR-k, and tau index compared with those in HFpEF-vehicle mice (*p* < 0.05, Table 2). Pulmonary congestion is also an iconic characteristic of HFpEF. We finally explored the effect of RES treatment on HFpEF-induced pulmonary congestion. As shown in Figure 2D, RES administered notably mitigated the HFpEF-induced pulmonary congestion. These results suggest that RES could ameliorate HFpEF-induced LVH, diastolic dysfunction, and pulmonary congestion.

**TABLE 2:** Echocardiography and invasive parameters of Sham or HFpEF mice 4 weeks after Vehicle or RES administration.

Parameter	Sham-vehicle (*n* = 8)	Sham-RES (*n* = 8)	HFpEF-vehicle (*n* = 8)	HFpEF-RES (*n* = 8)
*Echo parameters*				
LVESD (mm)	2.2 ± 0.5	2.4 ± 0.4	2.5 ± 0.6	2.4 ± 0.6
LVEDD (mm)	3.8 ± 0.4	3.8 ± 0.7	3.9 ± 0.9	4.0 ± 0.7
EF (%)	78 ± 5	79 ± 6	76 ± 8	77 ± 6
FS (%)	41 ± 5	42 ± 6	40 ± 6	40 ± 5
*Invasive parameters*				
Ves (ml)	25.0 ± 2.8	24.2 ± 2.8	23.8 ± 3.9	25.6 ± 4.2
Ved (ml)	36.1 ± 4.2	35.8 ± 4.7	37.0 ± 5.3	36.5 ± 4.9
Pes (mmHg)	129.6 ± 18.1	128.2 ± 20.5	127.9 ± 19.8	127.4 ± 18.9
Ped (mmHg)	7.7 ± 1.9	7.8 ± 2.1	7.9 ± 3.8	7.8 ± 3.5
EF (%)	67 ± 5	69 ± 8	65 ± 7	68 ± 6
dp/dtmax (mmHg/s)	14,160 ± 752.3	13,960 ± 685.9	14,380 ± 896.8	13,250 ± 953.8
dp/dtmin (mmHg/s)	−13,190 ± 153	−13,360 ± 353	−6,485 ± 168^a^	−10,587 ± 152^b^
Tau (ms)	5.5 ± 1.4	5.7 ± 1.5	9.0 ± 1.6^a^	7.3 ± 1.4^b^
EDPVR-k	1.81 ± 0.7	1.96 ± 0.9	7.34 ± 0.9^a^	4.03 ± 0.8^b^

Data are presented as mean ± SD. LVESD: left ventricular end systolic diameter; LVEDD: left ventricular end diastolic diameter; Ves: end-systolic volume; Ved: end-diastolic volume; Pes: end-systolic pressure; Ped: end-diastolic pressure; EF: ejection fraction; EDPVR: end-diastolic pressure–volume relationship; k: constant of an exponential curve fit of the EDPVR (a measure of active myocardial relaxation).

a p < 0.05 vs. sham-vehicle group.

b p < 0.05 vs. HFpEF-vehicle group.

### Resveratrol Decreased Heart Failure With Preserved Ejection Fraction–Induced Cardiac Inflammatory Response

**FIGURE 3:**
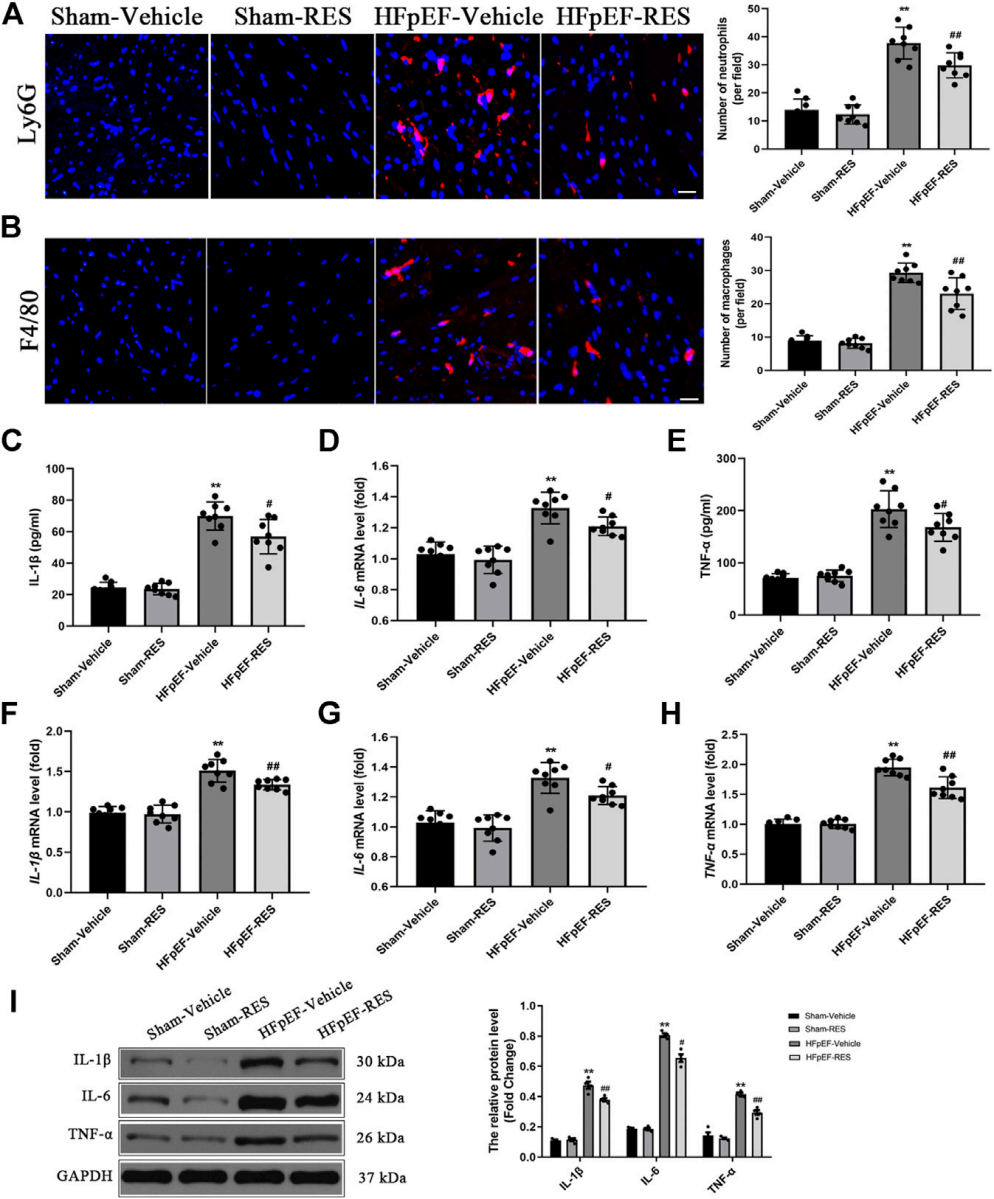
Effect of RES on HFpEF-induced inflammation. **(A)** Representative Ly6G and **(B)** F4/80 immunofluorescence staining. **(C–E)** Concentration of plasma pro-inflammatory cytokines IL-1β, IL-6, and TNF-α (*n* = 8 per group). **(F–H)** Results of IL-1β, IL-6, and TNF-α mRNA expressions (*n* = 8 per group). **(I)** Representative western blots and quantitative results of the IL-1β, IL-6, and TNF-α proteins (*n* = 8 per group). **p < 0.01 vs. sham-vehicle group. #*p* < 0.05 vs. HFpEF-vehicle group. ##*p* < 0.01 vs. HFpEF-vehicle group. Scale bars, 50 µm.

A pro-inflammatory state that leads to the pathophysiology of HFpEF has been recently emphasized (Paulus and Tschope, 2013; van Empel and Brunner-La Rocca, 2015; van Sanders-Wijk et al., 2015; Riba et al., 2017; Ratchford et al., 2019). We were interested in determining whether RES can decrease HFpEF-induced cardiac inflammatory response. As shown in Figures 3C–E, HFpEF mice showed markedly upregulated levels of plasma IL-1β, IL-6, and TNF-α than those in the sham-vehicle group (*p* < 0.05), and RES significantly decreased the release of HFpEF-induced pro-inflammatory cytokines (IL-1β, IL-6, and TNF-α) (*p* < 0.05, Figures 3C–E). Moreover, immunofluorescence staining also demonstrated increased infiltration of neutrophils and macrophages in the HFpEF hearts, and RES markedly reduced HFpEF-induced infiltration of neutrophils and macrophages (*p* < 0.05, Figures 3A,B). We then performed RT-PCR and western blot analysis to further determine the cardiac inflammation in HFpEF hearts. As shown in Figures 3F–H, the mRNA expression of IL-1β, IL-6, and TNF-α in HFpEF hearts significantly increased than that in sham-vehicle hearts (*p* < 0.05), and RES markedly decreased IL-1β, IL-6, and TNF-α mRNA expressions. Similar to the results of RT-PCR analysis, western blot analysis depicted increased IL-1β, IL-6, and TNF-α protein expressions in HFpEF hearts, and RES significantly decreased IL-1β, IL-6, and TNF-α protein expressions (*p* < 0.05, Figure 3I).

### Resveratrol Inhibited Macrophage Polarization in the Heart Failure With Preserved Ejection Fraction Hearts

**FIGURE 4:**
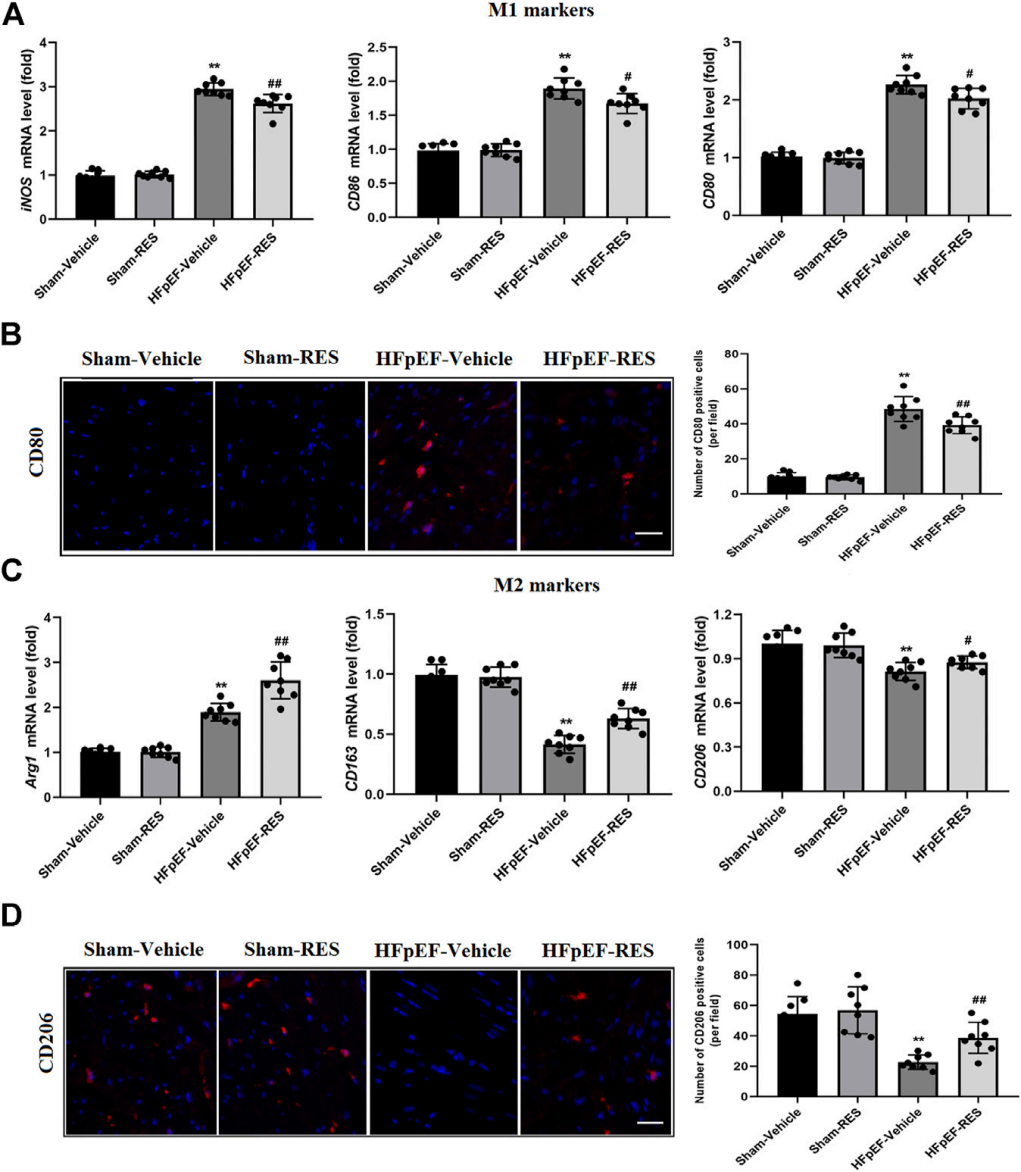
Effect of RES on HFpEF-induced macrophage polarization. **(A)** Results of mRNA expression of M1 markers *iNOS*, *CD86*, and *CD80* (*n* = 8 per group). **(B)** Representative CD80 immunofluorescence staining and quantitative results of the numbers of CD80-positive cells (*n* = 8 per group). **(C)** Results of mRNA expression of M2 markers *Arg1*, *CD163*, and *CD206* (*n* = 8 per group). **(D)** Representative CD206 immunofluorescence staining and quantitative results of the numbers of CD206-positive cells (*n* = 8 per group). *p < 0.05 vs. sham-vehicle group. **p < 0.01 vs. sham-vehicle group. #*p* < 0.05 vs. HFpEF-vehicle group. ##*p* < 0.01 vs. HFpEF-vehicle group. Scale bars, 50 µm.

Whether macrophage polarization is involved in the pathophysiological process of HFpEF remains unclear. The previous study reported that cardiac macrophages serve as therapeutic targets for cardiac fibrosis and could lead to HFpEF (Hulsmans et al., 2018). To check the effect of RES on macrophage differentiation in HFpEF, we firstly detected the mRNA expression of surface markers of macrophages in the heart by using qRT-PCR. The mRNA levels of M1 markers, including iNOS, CD86, and CD80, were obviously increased in the HFpEF-vehicle group, but treatment with RES significantly inhibited their increase induced by HFpEF (Figure 4A). Conversely, the HFpEF-vehicle group showed reduced mRNA expression of M2 markers (Arg1, CD163, and CD206), and RES administered markedly reversed this trend (Figure 4C). Moreover, immunofluorescence staining depicted similar results, increased CD80-stained immunofluorescence (Figure 4B) and decreased CD206-stained immunofluorescence (Figure 4D) were observed in HFpEF hearts than sham hearts, and RES could reverse this trend induced by HFpEF. Taken together, these results indicate that RES could offset HFpEF-induced inflammatory response and promote macrophage polarization toward the M2 phenotype.

### Resveratrol Reversed Heart Failure With Preserved Ejection Fraction–Induced Oxidative Stress

**FIGURE 5:**
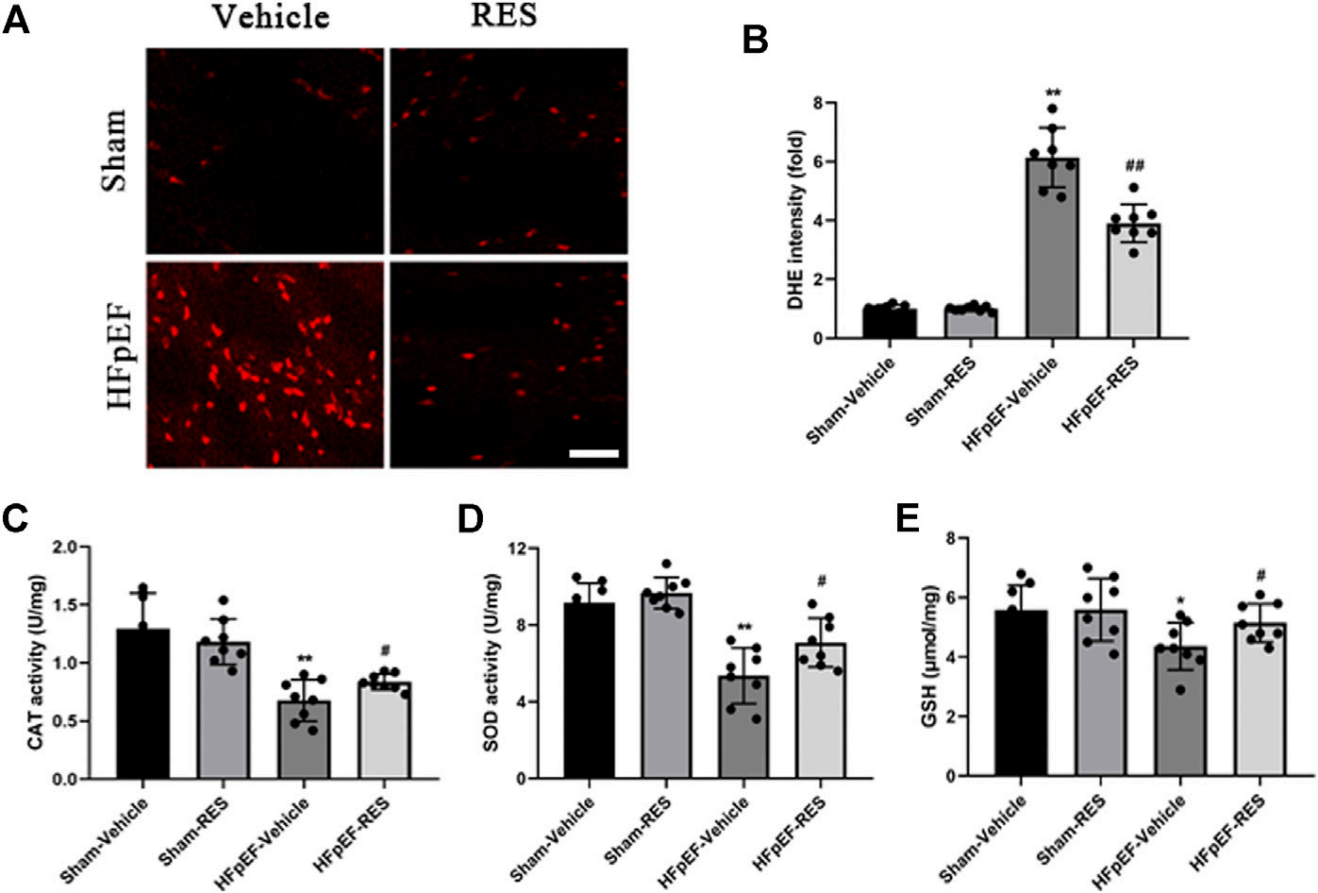
Effect of RES on HFpEF-induced oxidative stress. **(A)** Representative images and **(B)** quantitative results of DHE-stained heart sections (*n* = 8 per group). **(C)** Activities of CAT after RES treatment (*n* = 8 per group). **(D)** Activities of SOD after RES treatment (*n* = 8 per group). **(E)** Content of GSH after RES treatment (*n* = 8 per group). *p < 0.05 vs. sham-vehicle group. **p < 0.01 vs. sham-vehicle group. #*p* < 0.05 vs. HFpEF-vehicle group. Scale bars, 50 µm.

Excessive ROS formation is associated with systemic inflammatory response, which may cause an impaired cardiac function (Riba et al., 2017). Moreover, excessive ROS is associated with HFpEF (Vitiello et al., 2014; Liu et al., 2018; Yang et al., 2020). DHE staining and ELISA kits were used to measure ROS levels. As shown in Figures 5A,B, HFpEF hearts exhibited increased DHE-stained immunofluorescence than sham hearts (*p* < 0.05). In addition, further detection revealed increased activities of CAT, SOD, and GSH in HFpEF hearts compared with sham hearts (*p* < 0.05, Figures 5C–E). As expected, these pathological alterations were alleviated after RES treatment (*p* < 0.05, Figures 5C–E), which indicated RES to be an anti-oxidant factor in HFpEF.

### Resveratrol Attenuated Cardiac Fibrosis Induced by Heart Failure With Preserved Ejection Fraction

**FIGURE 6:**
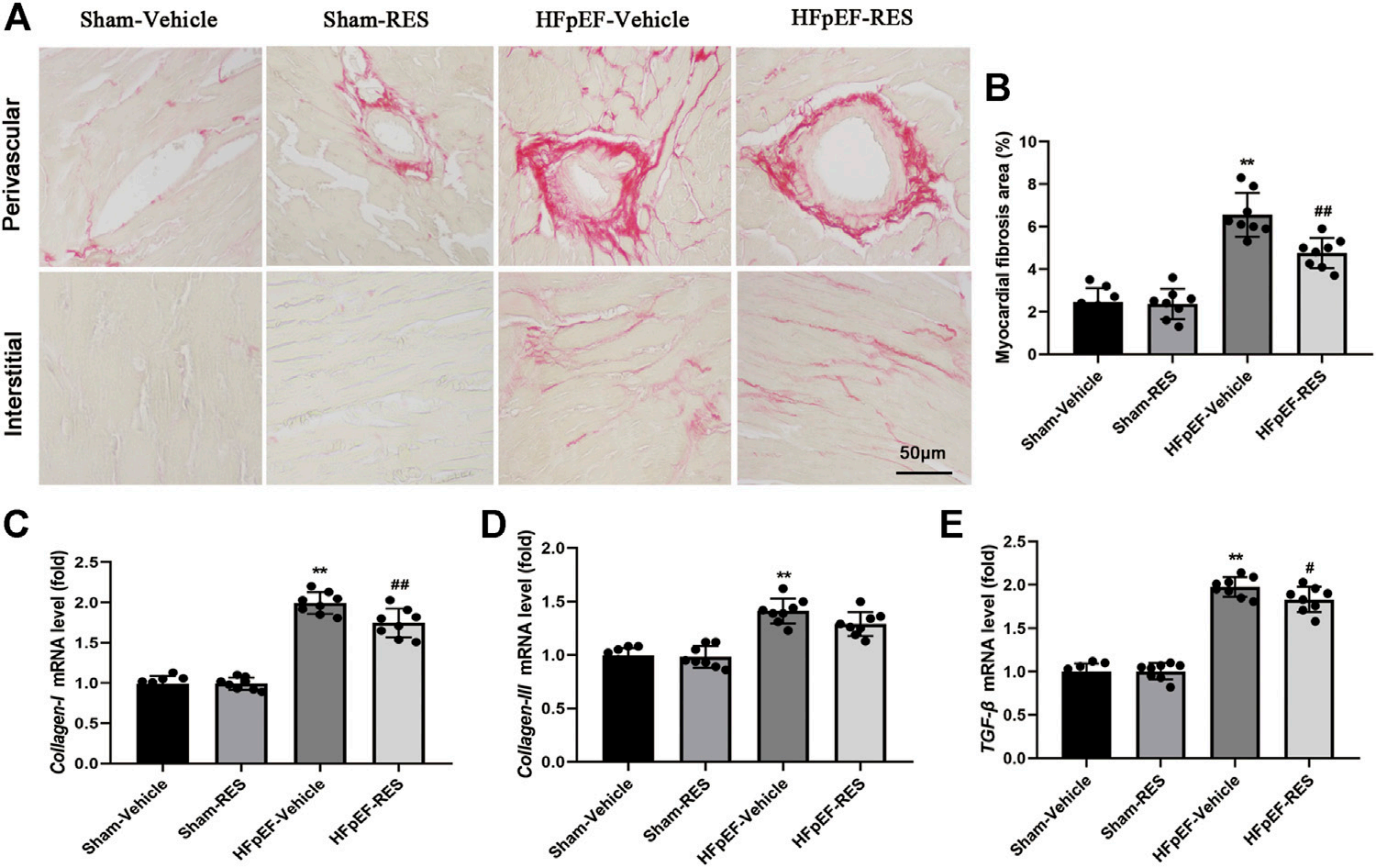
Effect of RES on HFpEF-induced cardiac fibrosis. **(A)** Representative images of picrosirius red (PSR)–stained heart sections and **(B)** quantitative results of fibrosis area. **(C–E)** Results of *collagen-I* and *-III* and TGF-β mRNA expressions (*n* = 8 per group). **p < 0.01 vs. sham-vehicle group. #*p* < 0.05 vs. HFpEF-vehicle group. ##*p* < 0.01 vs. HFpEF-vehicle group.

Cardiac fibrosis could cause cardiomyocyte stiffness, which was reported to play an important role in HFpEF (Sweeney et al., 2020). Picrosirius red (PSR) staining was then conducted to quantify cardiac fibrosis. Figure 6A shows representative perivascular and interstitial collagen of the four groups. After 4 weeks of RES treatment, collagen content in the HFpEF-vehicle group significantly increased when compared with that in the sham-vehicle group (*p* < 0.05, Figure 6B), and cardiac fibrosis induced by HFpEF was reduced when RES was administered (*p* < 0.05, Figure 6B). Furthermore, the mRNA expression of cardiac fibrosis markers *collagen-I* and *-III* and TGF-β significantly increased in the HFpEF-vehicle group compared with the sham-vehicle group (*p* < 0.05, Figures 6C–E), and these changes induced by HFpEF were attenuated when RES was administered (*p* < 0.05, Figures 6C–E).

### Resveratrol Alleviated Cardiac Stiffness Induced by Heart Failure With Preserved Ejection Fraction

**FIGURE 7:**
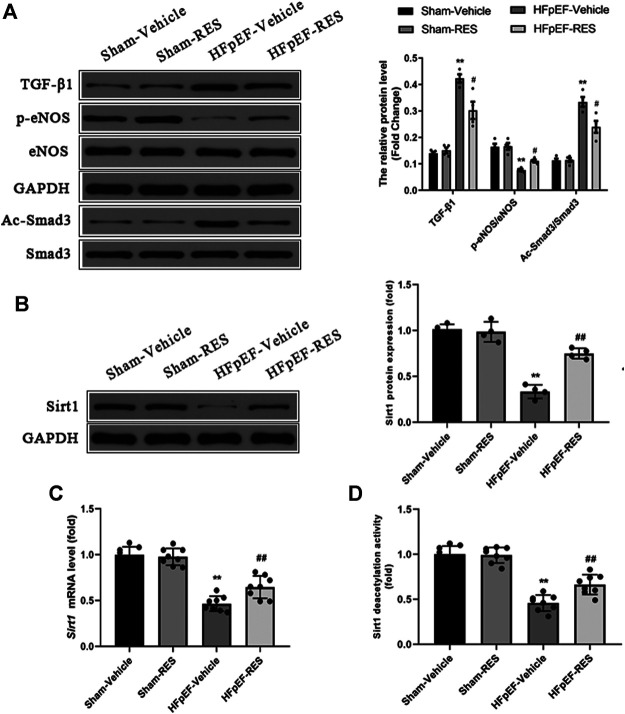
Effect of RES on the HFpEF-activated TGF-β/Smad3 signaling pathway and Sirt1 expression. **(A)** Representative western blots, immunoprecipitation, and quantitative results of the TGF-β1, Smad3, Ac-Smad3, p-eNOS, and eNOS proteins (*n* = 4 per group). **(B)** Representative western blots and quantitative results of the Sirt1 proteins (*n* = 4 per group). **(C)** Quantitative results of *Sirt1* mRNA (*n* = 8 per group). **(D)** Quantitative results of Sirt1 deacetylation activity (*n* = 8 per group). **p < 0.01 vs. sham-vehicle group. #*p* < 0.05 vs. HFpEF-vehicle group.

Decreased cardiac eNOS expression can cause endothelial function, leading to cardiac stiffness and ultimately HFpEF (Wilck et al., 2018). Besides, RES could also rescure decreased eNOS expression in a post-infarction heart failure model (Riba et al., 2017). Hence, we evaluated whether RES could alleviate HFpEF-induced cardiac stiffness. Western blot exhibited decreased protein expression of phosphorylated eNOS in HFpEF hearts, and RES markedly increased phosphorylated eNOS expression (*p* < 0.05, Figure 7A).

### Resveratrol Inhibited the Activation of the Transforming Growth Factor-β/Smad3 Signaling Pathway in Heart Failure With Preserved Ejection Fraction

The TGF-β/Smad3 signaling pathway is a well-known signaling pathway that regulates cardiac fibrosis. Increased acetylated-Smad3 (Ac-Smad3) protein expression is associated with increased cardiac fibrosis (Zhang et al., 2017; Li et al., 2018), and RES was reported to decrease the Ac-Smad3 protein expression to inhibit renal fibrosis (Huang et al., 2014). We then sought to assess whether the TGF-β/Smad3 signaling pathway contributes to the protective effect of RES on HFpEF-induced cardiac fibrosis and evaluate the effect of RES on the cardiac Ac-Smad3 protein expression. As shown in Figure 7A, the protein levels of TGF-β1 and Ac-Smad3 in the HFpEF group were significantly elevated compared to those in the sham group, and RES markedly inhibited the activation of the TGF-β/Smad3 signaling pathway by decreasing the acetylated-Smad3 protein expression in HFpEF hearts (*p* < 0.05, Figure 7A).

### RES Inhibited Smad3 Acetylation by Activating Sirtuin 1

**FIGURE 8:**
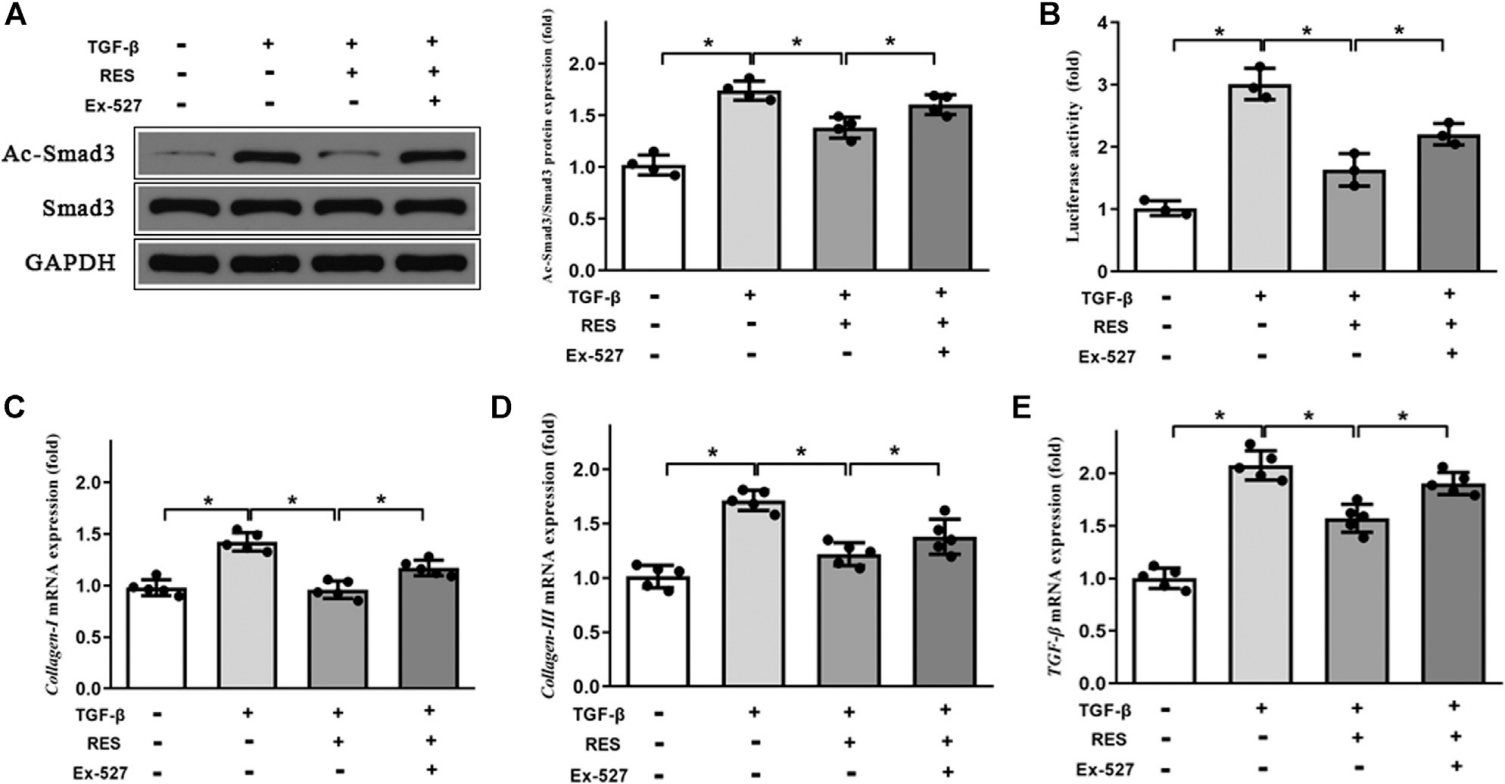
Effect of Sirt1 inhibition on the Smad3 expression and pro-fibrotic markers’ mRNA expression in CFs. **(A)** Representative western blots and quantitative results of the Smad3 and Ac-Smad3 proteins in CFs (*n* = 4 per group). **(B)** The transcriptional activity of SMAD3 is represented as the intensity of luciferase reporter gene activity (*n* = 3 per group). Quantitative results of mRNA expression of the pro-fibrotic markers **(C)** *collagen-I*, **(D)** *collagen-III*, and **(E)** TGF-β in CFs (*n* = 5 per group). **p < 0.05.

The previous study reported that RES is a Sirt1 activator (Ma et al., 2017). We assessed the effect of RES on Sirt1 expression in HFpEF. As shown in Figures 7B,C, HFpEF mice showed decreased Sirt1 protein and mRNA expression compared to Sham mice. RES administered significantly increased Sirt1 protein and mRNA expression compared to HFpEF mice. Then we evaluated effect of RES on Sirt1 deacetylation activity, as shown in Figure 7D, HFpEF mice exhibited decreased Sirt1 deacetylation activity compared to Sham mice. RES administered significantly increased Sirt1 deacetylation activity compared to HFpEF mice. Moreover, activated Sirt1 was reported to inhibit the activation of the TGF-β/Smad3 signaling pathway by decreasing the acetylated-Smad3 protein expression (Ma et al., 2017; Zhang et al., 2017; Li et al., 2018). To further determine whether RES exerted its protective effect by regulating Smad3, we sought to determine the levels of acetylated and total Smad3 protein expressions in CFs. The ratios of Ac-Smad3/Smad3 were significantly increased in TGF-β1–stimulated CFs, and treatment with RES significantly decreased the ratios of Ac-Smad3/Smad3. We then co-treated CFs with Ex-527, TGF-β, and RES and found that Ex-527 offset the effect of RES on TGF-β1–induced increased Ac-Smad3 protein expression in CFs (Figure 8A). Smad3 transcriptional activity is weakened when it is deacetylated. To examine the Smad3 transcriptional activity, the luciferase reporter assay was used. As shown in Figure 8B, inhibiting Sirt1 with Ex-527 substantially enhanced TGF-β1–induced Smad3-dependent transcriptional activity. RES treatment significantly reduced TGF-β1–induced Smad3-dependent transcriptional activity, which indicated that RES with Ex-527 diminished TGF-β1–induced Smad3 transcriptional activity and inhibited Sirt1-enhanced Smad3-dependent transcriptional activity. Finally, we examined the role of Sirt1 in the effect of RES on TGF-β–induced cardiac fibrosis *in vitro*. Ex-527 was used to decrease the Sirt1 expression. As shown in Figures 8C–E, the mRNA expression of cardiac fibrosis markers *collagen-I* and *-III* and TGF-β was significantly increased when treating CFs with TGF-β; co-treating CFs with TGF-β and RES significantly decreased the mRNA expression of cardiac fibrosis markers *collagen-I* and *-III* and TGF-β. And inhibited Sirt1 with Ex-527 co-treated with TGF-β and RES offset the protective role of RES in TGF-β–induced cardiac fibrosis.

## Discussion

**FIGURE 9:**
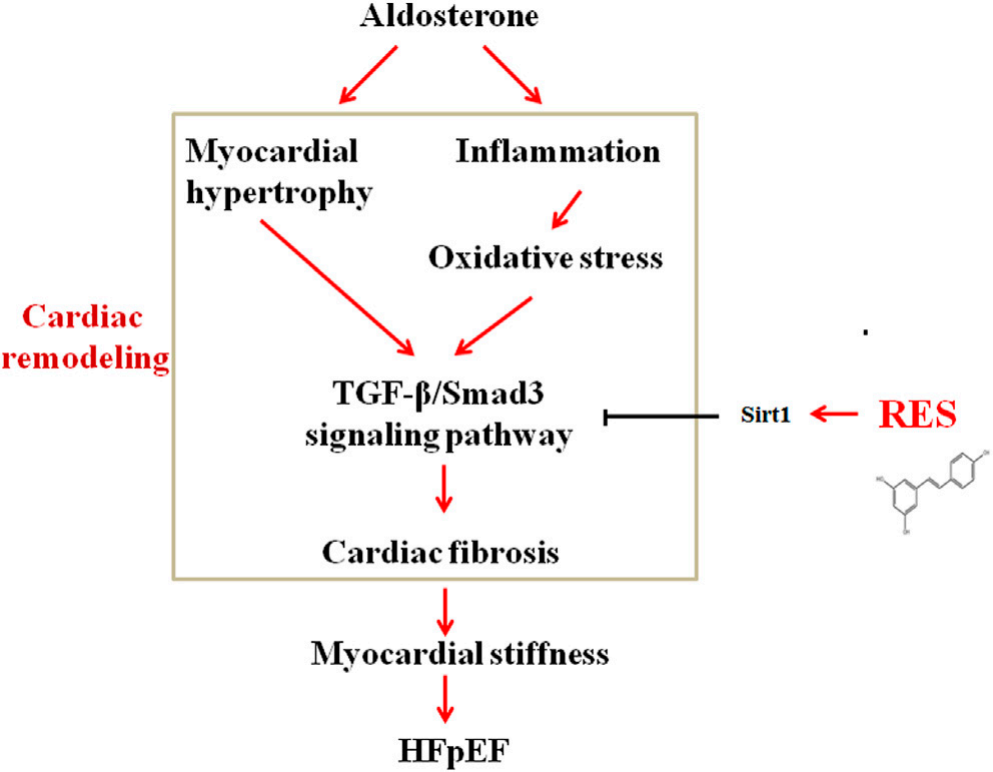
Schematic diagram showing possible mechanisms by which aldosterone induces cardiac fibrosis in HFpEF.

This study mechanistically explored the effect of RES on cardiac remodeling in a murine model of HFpEF. The novel findings of the present study are as follows: 1) RES could significantly ameliorate HFpEF-induced LVH, diastolic dysfunction, and pulmonary congestion without impairing the LVEF; 2) RES treatment decreased the infiltration of inflammatory cells and regulated M1/M2 macrophage polarization in the heart; 3) RES reversed HFpEF-induced superoxide production, restored HFpEF-induced extracellular matrix deposition, and increased the phosphorylated eNOS expression; 4) in addition, RES also inhibited the TGF-β/Smad3 pro-fibrotic signaling pathway; 5) RES decreased Smad3 acetylation and inhibited Smad3 transcriptional activity *via* activating Sirt1; and 6) inhibited Sirt1 with Ex-527 offset the protective role of RES in TGF-β–induced cardiac fibroblast–myofibroblast transformation. In summary, these findings indicate that RES might be a potent drug that exerts protective effect on HFpEF-induced cardiac remodeling by inhibiting inflammatory responses, modulating macrophage polarization, reversing oxidation, activating Sirt1, and suppressing Smad3 acetylation, thereby reducing cardiac fibrosis and cardiac stiffness (Figure 9). These observations add further evidence to the molecular understanding of the treatment of RES on HFpEF.

Accumulating evidence suggested that RES exerts anti-inflammatory effect on several settings of cardiovascular diseases except HFpEF. This time, we aimed to test the cardioprotective effect of RES on cardiac inflammation and the potential mechanisms. Previous experiments revealed that myocardial inflammatory response plays a pivotal role in the development of HFpEF. In an animal experiment, circulating levels of the pro-inflammatory cytokines TNF-α, IL-6, and IL-1β were markedly increased in HFpEF mice compared to sham mice (Shuai et al., 2020). Furthermore, HFpEF patients showed higher plasma inflammatory cytokines IL-6 and TNF-α than healthy population (Collier et al., 2011). A systemic pro-inflammatory state induced by HFpEF results in coronary microvascular endothelial inflammation, causing reduced nitric oxide (NO) bioavailability and decreasing cyclic guanosine monophosphate (cGMP) content and protein kinase G (PKG) activity, leading to endothelial dysfunction (Paulus and Tschope, 2013). In line with previous reports, aldosterone-induced HFpEF mice in the current study exhibited higher levels of plasma IL-1β, IL-6, and TNF-α than sham mice (Figure 3). And RES administered significantly decreased HFpEF-induced inflammatory response, indicating RES also has an anti-inflammatory property in the HFpEF setting.

Inappropriate macrophage polarization and excessive inflammatory factors are reported to be linked with increased macrophage infiltrates in the impaired myocardium (Glezeva et al., 2015). However, whether macrophage polarization is involved in the pathophysiological process of HFpEF remains unclear even though RES was reported to promote the M1-to-M2 macrophage transition (Yu et al., 2019; Shabani et al., 2020). Cardiac macrophages could produce pro-inflammatory cytokines, which may activate fibroblasts, stimulate collagen deposition, lead to impaired myocardial relaxation, increase myocardial stiffness, and eventually contribute to HFpEF (Hulsmans et al., 2018). So, we evaluated the effect of RES on the HFpEF mice model, and interestingly, in the current study, we found that RES promoted the M1-to-M2 macrophage transition as evidenced by the decreased mRNA levels of M1 markers, including iNOS, CD86, and CD80, and increased mRNA levels of M2 markers Arg1, CD163, and CD206. Thus, it can be speculated that RES modulates cardiac inflammation in HFpEF mice by modulating macrophage polarization.

Excessive myocardial ROS release plays a vital role in HFpEF. It is likely a systemic inflammatory state that can both produce free radicals and blunt cellular anti-oxidant capacity. Moreover, excessive ROS could lead to collagen uncoupling and the formation of superoxide anions, reduce NO bioavailability, and ultimately impair cardiac function (Cosentino and Luscher, 1999). It was reported that a significant increase in biomarkers is related to oxidative stress including thiobarbituric acid reactive substances (TBARS) and 8-epi-prostaglandin F2α in HFpEF patients compared with healthy subjects, confirming a pro-oxidative state in these patients (Vitiello et al., 2014). The animal model of HFpEF also exhibited increased ROS production as shown by increased SOD1 and SOD2 protein expressions (Yang et al., 2020). In the present study, the HFpEF mice depicted increased activities of CAT, SOD, and GSH, which were further verified by DHE staining, and indicated an oxidative state in HFpEF hearts, which is parallel to that previously reported (Yang et al., 2020). Abundant evidence showed that RES exerted protective effect on several cardiovascular diseases (Wojciechowski et al., 2010; Dolinsky et al., 2013). It is not a surprise that RES also exerted anti-oxidative effect on HFpEF hearts. In this study, RES markedly reduced activities of CAT, SOD, and GSH induced by HFpEF, which further verified an anti-oxidative role of RES not only in heart failure with reduced ejection fraction (HFrEF) but also in the setting of HFpEF.

The NO-cGMP-PKG pathways were reported to be a potentially promising therapeutic target for HFpEF (Greene et al., 2013; Pieske et al., 2014). Cardiac inflammation and oxidative stress in HFpEF cause decreased levels of eNOS, as a consequence of diminished NO bioavailability (van Heerebeek et al., 2012). As NO contributes to myocardial relaxation, reduced cardiac eNOS contributes to diastolic dysfunction (Takimoto et al., 2005). In the current study, HFpEF mice showed decreased protein expression of p-eNOS; treatment with RES counteracted eNOS uncoupling and alleviated myocardial stiffness in HFpEF mice.

The inflammation-driven stimulation of TGF-β/Smad3 signaling is essential for the transdifferentiation of fibroblasts into myofibroblasts and collagen deposition (Esposito et al., 2017). Furthermore, oxidative stress could also activate the TGF-β/Smad3 signaling pathway which eventually leads to cardiac fibrosis (Kong et al., 2020). Excessive collagen deposition could cause cardiomyocyte stiffness, which is the main manner of cardiac remodeling in HFpEF. In HFpEF hearts, cardiac fibroblasts are presumed to convert to myofibroblasts because of exposure to TGF-β as a result of macrophage myocardial infiltration. In our study, both protein levels of TGF-β and its downstream effector Smad3 decreased following RES treatment. Furthermore, we detected a reduction of cardiac fibrosis in HFpEF when treated with RES. It is well-documented that Smad3 acetylation is a key signaling mechanism underlying fibrogene in response to TGF-β treatment (Ma et al., 2017; Zhang et al., 2017; Li et al., 2018). And RES could significantly reduce the Smad3 acetylation levels in the remnant kidney of 5/6 nephrectomized rodents or in cultured cells following TGF-β1 treatment–induced renal fibrosis (Huang et al., 2014). Similar to Huang et al.’s (2014) report, the current study also found increased Smad3 acetylation in HFpEF hearts and in cultured TGF-β1–treated CFs; RES significantly reduced Smad3 acetylation in HFpEF hearts and in cultured TGF-β1–treated CFs.

Sirtuin 1 (Sirt1) deacetylase is a class deacetylase, which could deacetylate various proteins (Li et al., 2018). Emerging studies reported that decreased Sirt1 deacetylase activity and protein expression were found in hearts under pathological stimulation and activated Sirt1 could serve as a protective agent against cardiac fibrosis by decreasing Smad3 acetylation and reducing Smad3 transcriptional activity in hearts (Cappetta et al., 2016; Ma et al., 2017; Li et al., 2018; Liu et al., 2019). In addition, RES is a well-known Sirt1 activator (Huang et al., 2014; Liu et al., 2019). Based on these studies, we detected Sirt1 mRNA, protein expression, and deacetylase activity in HFpEF hearts and TGF-β–treated CFs. As expected, Sirt1 protein, mRNA expression, and deacetylase activity in HFpEF mice and TGF-β–treated CFs were significantly decreased, resulting in increased levels of Smad3 acetylation. RES administered markedly increased Sirt1 mRNA, protein expression, and deacetylase activity, resulting in decreased levels of Smad3 acetylation, which suggested that decreased Sirt1 expression leads to reduced deacetylase activity of Sirt1 and increased levels of Smad3 acetylation in HFpEF mice and TGF-β–treated CFs. The process mentioned above could be reversed by RES administration. Inhibited Sirt1 with Ex-527 co-treated with TGF-β and RES offset the protective role of RES in TGF-β–induced increased Smad3 acetylation and cardiac fibrosis, which further verified the effect of RES on Sirt1/Smad3 interaction in the setting of HFpEF.

Thus, this study provides evidence that HFpEF results in a systemic pro-inflammatory state, causes excessive ROS production, leads to downregulation of eNOS and myocardial stiffness, inhibits Sirt1 activation, increases Smad3 acetylation, activates the TGF-β signaling pathway, and eventually enhances myocardial fibrosis (Figure 9). By inhibiting the chain of events, RES exerted a beneficial modulation of cardiac remodeling induced by HFpEF.

## Conclusion

In conclusion, it is clear that the mechanisms responsible for the beneficial effects of RES on HFpEF-induced cardiac remodeling are completely complex and various. The present study provides novel insights into the fact that the nutritional agent RES may exert protective effect in the setting of HFpEF by combining anti-inflammatory, anti-oxidant, and anti-fibrotic actions coupled with improved cardiac stiffness.

## Data Availability

The raw data supporting the conclusions of this article will be made available by the authors, without undue reservation.

## Supplementary Material

DATA SHEET 1 Raw datas used in this manuscript.

DATA SHEET 2 Original western blots of Figures 3, 7, and 8.
