# Corrigendum: Resveratrol Ameliorates Cardiac Remodeling in a Murine Model of Heart Failure With Preserved Ejection Fraction

**DOI:** 10.3389/fphar.2022.857367

**Published:** 2022-03-18

**Authors:** Liyun Zhang, Juan Chen, Lianhua Yan, Qin He, Han Xie, Manhua Chen

**Affiliations:** Department of Cardiology, The Central Hospital of Wuhan, Tongji Medical College, Huazhong University of Science and Technology, Wuhan, China

**Keywords:** resveratrol, heart failure with preserved ejection fraction, inflammation, macrophage polarization, oxidative stress

In the original article, there were two errors. The Wuhan Central Hospital’s animal experiment center was not involved in providing the animals nor in approving experimental procedures, but rather the animal experiment center of Tongji Medical College, Huazhong University of Science and Technology.

A correction has been made to **Materials and Methods**, “Animals,” paragraph 1. The corrected paragraph appears below:

“Male C57BL/6 mice (8- to 10-week-old) were supplied by the animal experiment center of Tongji Medical College, Huazhong University of Science and Technology. Mice were allowed 1 week to acclimatize to a stable environment before experiments began. Mice were individually housed in plastic cages with bedding, ad libitum food, and tap water. The cages were maintained at a temperature of 22 ± 2°C and a 12:12 h light/dark cycle. Mice were randomly assigned to four groups: sham with vehicle (sham-vehicle), sham with RES (sham-RES), HFpEF with vehicle (HFpEF-vehicle), and HFpEF with RES (HFpEFRES). All mice underwent uninephrectomy and received a continuous infusion of either saline (sham) or d-aldosterone (0.15 mg/h) (HFpEF) for 4 weeks *via* osmotic mini-pumps (Alzet, Durect Corp., Cupertino, CA, United States) (**Tanaka et al., 2014**). Twenty-4 hours after the surgery, mice were administered RES (10 mg/kg/day) by oral gavage for the duration of 4 weeks (**Gupta et al., 2014**) (**Figure 1B**). All experimental procedures were according to the Guidelines for the Care and Use of Laboratory Animals published by the United States National Institutes of Health and were approved by the Animal Care and Use Committee of Tongji Medical College, Huazhong University of Science and Technology (Approval Number: 20191808).”

The authors apologize for this error and state that this does not change the scientific conclusions of the article in any way. The original article has been updated.

