# Supplementary figures and images for: Resveratrol Ameliorates Cardiac Remodeling in a Murine Model of Heart Failure With Preserved Ejection Fraction
Source: Front Pharmacol. 2021 Jun 10;12:646240. doi: 10.3389/fphar.2021.646240 (PMC8225267; doi:10.3389/fphar.2021.646240)

**Figure 3**

**
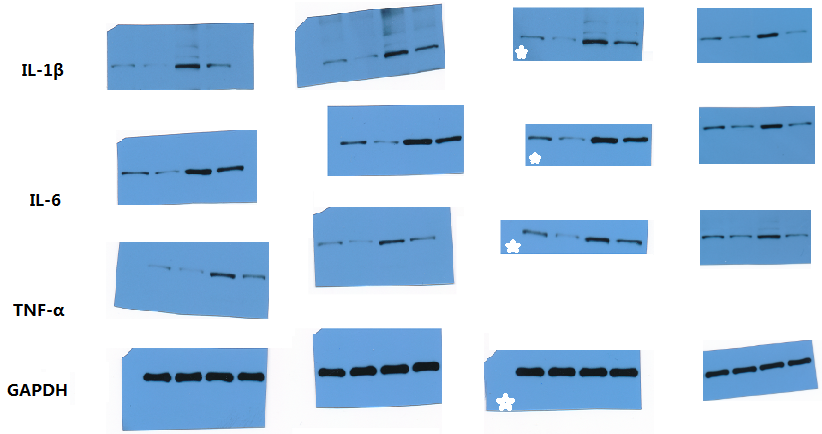
**

**Figure 7**

**
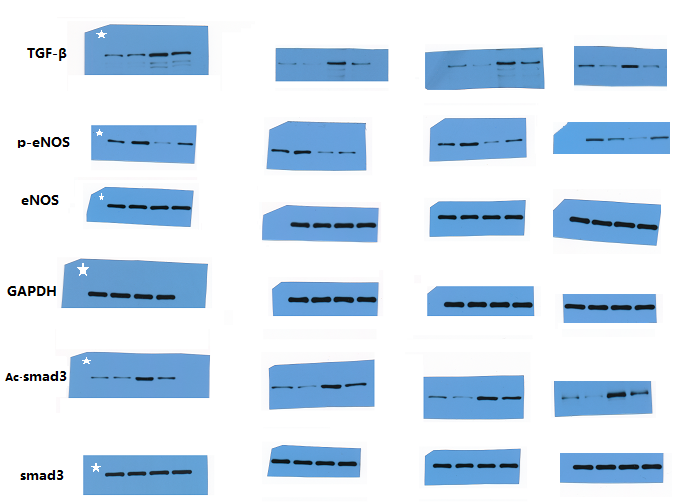

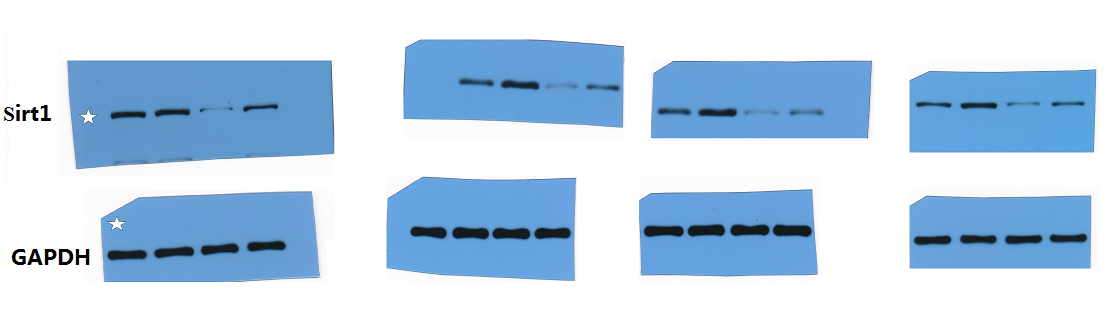
**

**Figure 8**

**
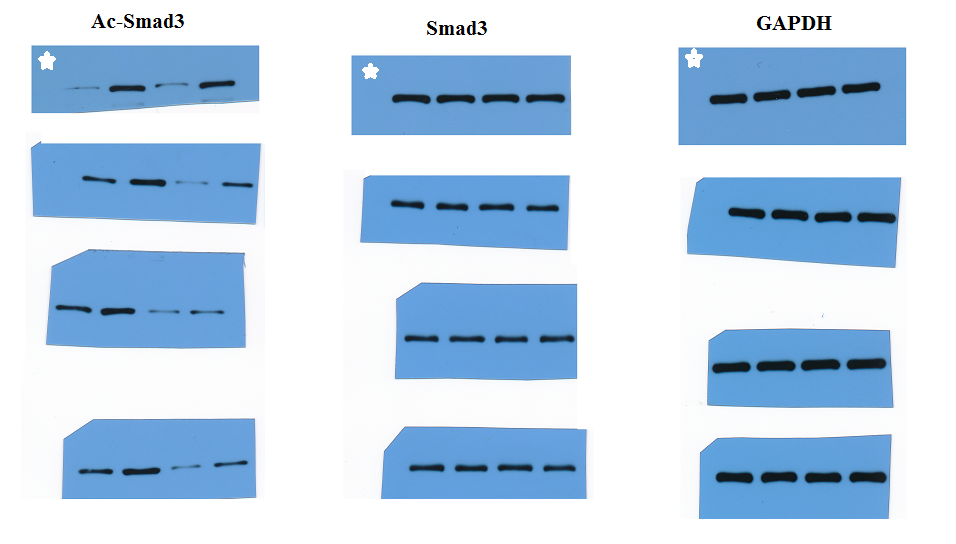
**

Supplement: Supplementary file 1 [file DataSheet1.doc]
